# Effects of alcohol mixed with energy drink and alcohol alone on subjective intoxication

**DOI:** 10.1007/s00726-013-1603-0

**Published:** 2013-11-01

**Authors:** Andrea Ulbrich, Sophie Helene Hemberger, Alexandra Loidl, Stephanie Dufek, Eleonore Pablik, Sugarka Fodor, Marion Herle, Christoph Aufricht

**Affiliations:** 1 Department of Pediatrics and Adolescent Medicine, Medical University of Vienna, Währinger Gürtel 18-20, 1090 Vienna, Austria; 2 Faculty of Psychology, University of Vienna, Liebiggasse 5, 1010 Vienna, Austria; 3 Institute of Medical Statistics, Medical University of Vienna, Spitalgasse 23, 1090 Vienna, Austria

**Keywords:** Energy drink, Red bull, Alcohol, Caffeine, Masking, Intoxication

## Abstract

Recent studies suggest that the combination of caffeine-containing drinks together with alcohol might reduce the subjective feelings of alcohol intoxication—the so-called “masking effect”. In this study, we aimed to review the effects of alcohol in combination with caffeine or energy drink with special focus on the “masking effect”. Fifty-two healthy male volunteers were analysed concerning breath alcohol concentration and subjective sensations of intoxication using a 18 item Visual Analogue Scale in a randomised, double-blinded, controlled, four treatments cross-over trial after consumption of (A) placebo, (B) alcohol (vodka 37.5 % at a dose of 46.5 g ethanol), (C) alcohol in combination with caffeine at a dose of 80 mg (equivalent to one 250 ml can of energy drink) and (D) alcohol in combination with energy drink at a dose of 250 ml (one can). Primary variables were headache, weakness, salivation and motor coordination. Out of four primary variables, weakness and motor coordination showed a statistically significant difference between alcohol and non-alcohol group, out of 14 secondary variables, five more variables (dizziness, alterations in sight, alterations in walking, agitation and alterations in speech) also showed significant differences due mainly to contrasts with the non-alcohol group. In none of these end points, could a statistically significant effect be found for the additional ingestion of energy drink or caffeine on the subjective feelings of alcohol intoxication. This within-subjects study does not confirm the presence of a “masking effect” when combining caffeine or energy drink with alcohol.

## Introduction

So-called “energy drinks”—usually based on caffeine, carbohydrates, vitamins and other ingredients such as taurine—have become popular and capture one percent of the total soft drink market. Since 2004, energy drinks have been the fastest growing sector of the beverage market, for example the market in Western Europe has grown by 12.9 % between 2007 and 2011 (Database 2012). One of the most popular energy drinks is Red Bull^®^, which has been available in Austria since 1987 and in the United States since 1997 (Reissig et al. 2009). It is now available in more than 160 countries.

The ingestion of caffeine-containing drinks together with alcohol is not a new phenomenon (e.g. rum coke/cola), and recently the combination of energy drinks with alcohol has become popular, with 20 % of students occasionally combining energy drinks with alcohol (de Haan et al. 2012). In the past, concerns have been raised suggesting that the combination of both substances might reduce the subjective feelings of alcohol intoxication—the so-called “masking effect” (Ferreira et al. 2006). Therefore the risk for dangerous activities, such as driving a car, would be increased. Thus possible effects of combined alcohol and caffeine consumption are an important research topic.

Several studies have already investigated whether caffeine counteracts the neuro-cognitive effects of alcohol consumption, with inconsistent results (Ferreira et al. 2006; Alford et al. 2012; Azcona et al. 1995; Marczinski et al. 2011). Some studies report a significant antagonising effect of caffeine on alcohol such as influencing weakness and impairment of motor coordination (Ferreira et al. 2006), or they suggest that the combination of caffeine and alcohol may lead to longer drinking and to an increase in stimulation compared to alcohol-only consumption (Attwood et al. 2012). Other investigations could not find any antagonising effects (Alford et al. 2012; Marczinski et al. 2012; Verster et al. 2012).

One of the most cited studies regarding the so-called “masking effect” was performed by (Ferreira et al. 2006). In this study, twenty-six young and healthy male volunteers were tested concerning breath alcohol concentration, subjective sensations of intoxication, motor coordination, and visual reaction time after consumption of energy drink (3.57 ml/kg bw), alcohol (0.6 or 1.0 g/kg bw) or both, using a mixed design. The additional ingestion of energy drinks did not modify breath alcohol concentration, motor coordination, and visual reaction time. Regarding subjective sensations of intoxication, more descriptors registered similar impairment with the energy drink and alcohol combination versus alcohol alone, than showed reduced impairment after co-administration of energy drink with alcohol. However, the authors interpreted these findings as a so-called “masking effect”, since they found significant differences in headache, weakness, salivation and motor coordination.

Given the variability of results regarding the perception of impairment, no valid evidence can be derived from this study. Therefore, the results and conclusion of this study should be re-assessed and discussed again, especially concerning the importance of the four mentioned subjective effects of intoxication (headache, weakness, salivation and motor coordination) which were reported to be significant. Since the publication of this study, several methodological flaws of this study have been discussed, in particular the statistical analysis, and the interpretation of the results are not undisputed (Alford et al. 2012; Verster et al. 2012). However, the data have neither been confirmed nor rebutted, and in a review published in 2012, Verster et al. conclude that the masking effect of energy drinks cannot be confirmed by currently available data (Verster et al. 2012).

In this study, we aimed to replicate Ferreira’s study with a higher number of healthy participants and a within-subjects design to re-examine the effects of alcohol in combination with caffeine or energy drink with special focus on the so-called “masking effect”.

## Methods

### Subjects

Fifty-two healthy male volunteers participated in the study. Their age was 20–26 years (24.4 ± 1.5), with body mass indices between 21 and 25 kg/m^2^ (23.2 ± 1.1), body weight between 68 and 85 kg (77.0 ± 3.8) and at least 12–14 years of formal education. All volunteers were in good general health as determined by medical history and screening investigations. All were taking no regular medication and had no history of psychiatric disorders.

Further inclusion criteria were: Moderate alcohol consumption (less than 190.4 g/week) according to the Daily Drink questionnaire (Collins et al. 1985), sporadic users of energy drinks (less than ten cans of 250 ml in the last 6 months), and confirmation from the participant’s general practitioner that they do not know of any reason that would advise against participation in the study. Participants were similar regarding social and demographic data, patterns of use of alcoholic beverages and energy drinks as well as quality of life (Martinez et al. 2000) and having a similar level of physical activity (Baecke et al. 1982). Exclusion criteria were a consumption of less than two or more than four caffeine-containing drinks per day within 3 months of screening; smoking of more than ten cigarettes per day or equivalent within 3 months of screening; consumption of more than 190.4 g alcohol per week; a history of alcohol or drug abuse or consumption of less than one alcoholic drink per week.

Volunteers were informed about the procedures of the study and signed an informed consent form. The Committee of Ethics of the Medical University of Vienna approved the study (ClinicalTrials.gov Identifier: NCT01350089).

### Treatments

Four mixtures listed below were consumed orally within 10–20 min, on one occasion each in a randomised order. Volunteers wore a nose clip to optimise blinding. As far as possible all investigational products were identical in appearance and taste, differing only in the absence/presence of alcohol, caffeine and energy drink. The final volume of the mixtures was 500 ml for each treatment. The caffeine, alcohol and energy drink doses were chosen as used by Ferreira et al. because they were within the range of doses usually ingested on a single occasion (Ferreira et al. 2006).

The placebo (A) consisted of (carbonated) water (250 ml), artificial fruit juice [21 g/l prepared with (carbonated) water].

The comparator B was a mixture of 46.5 g ethanol (in form of vodka 37.5 vol %) (carbonated) water (250 ml), artificial fruit juice [21 g/l prepared with (carbonated) water].

The comparator C was a mixture of 46.5 g ethanol (in form of vodka 37.5 vol %), caffeine (80 mg, equivalent to one 250 ml can of a typical energy drink), (carbonated) water (250 ml), artificial fruit juice [21 g/l prepared with (carbonated) water].

The comparator D was a mixture of 46.5 g ethanol (in form of vodka 37.5 vol %), Red Bull Energy Drink (250 ml, equivalent to one can, without flavour to optimise blinding), artificial fruit juice [21 g/l prepared with (carbonated) water].

### Subjective effects of intoxication

This was evaluated through a visual analogue scale (VAS) of somatic symptoms (Bond and Lader 1972; Greenwood et al. 1975) including all items used in Ferreira et al. 2006. This was assessed before and 30, 75 and 120 min after the treatments. Each 100 mm line represented the whole range of possible intensity of each listed symptom (e.g. salivation–dry mouth). The volunteers marked the location that corresponded to the intensity of their sensation with a vertical line. We tested the items: agitation, alterations in motor coordination, hearing, walking and speech, sensation of well-being, tiredness, headache, dizziness, tremor, weakness, muscular tension, nausea, salivation, perspiration, visual disturbances, tachycardia and difficulty in breathing.

### Procedures

The volunteers were instructed to drink no alcohol or high-energy products during each study period with the exception of study treatment. Sufficient sleep (at least 7 h) was required the night before testing and controlled via questionnaire on test days. No alcohol during a period of at least 72 h prior to each test dose was allowed. On the test days, the consumption of at least two and no more than four caffeine-containing drinks was controlled via questionnaire. On every treatment day, the volunteers were instructed to arrive fasting 15 min before the beginning of the treatment administration, which started around midday. A standard meal of 1,000 kcal (1 Big Mac, small French fries and water) was given 45 min before treatment. Sugar-free fluid was allowed until 1 h before treatment, no further fluids were allowed until 2.5 h after dosing. We used a double blind procedure throughout the experiment.

#### Safety

Supine and standing vital signs were evaluated before (in triplicate) and 60 and 150 min after the treatments.

### Breath alcohol concentration

This was determined by using a breath analyzer (AlcoQuant 6,020, Envitec, Germany) before and 15, 30, 60, 90, 120 and 150 min after the treatments. The alcohol dose used aimed for a mean breath alcohol concentration of 0.05 % similar to that of Ferreira et al. (2006).

### Statistical analyses

To check for differences between the four treatment groups mixed models were applied to consider the special data structure of the cross-over design. A restricted maximum likelihood (REML) method was used. The primary variables were the symptoms headache, weakness, salivation and motor coordination, as they yielded statistically significant differences in Ferreira’s study. To adjust for multiple comparisons a Bonferroni correction was applied for the four primary variables resulting in a local significance level of 0.05/4 = 0.0125 for each single primary variable.

In a first step the data of all four treatment groups were used. As the descriptive plots partially revealed considerable differences between the alcohol groups and the non-alcohol group, a further analysis was performed using only the three treatment groups that included alcohol.

In each of the models, the 20 min pre-treatment values of the respective treatment day were included as baseline values. The following measurements at 30, 90 and 120 min post-treatment were treated as autoregressive. We assumed, that due to the long wash-out period between the treatments no carry-over effect could occur, but in order to account for possible habituation effects the number of the visit was included in the model. Therefore, the fixed effects included in the model were baseline, treatment, number of the visit, time of measurement, and the interaction between treatment and time of measurement. A random influence of each patient was included in the model.

## Results

### Breath alcohol concentration

Mean breath alcohol concentrations at 15, 30, 60, 90, 120 and 150 min after the treatments were 0.059, 0.059, 0.053, 0.047, 0.041 and 0.035 %,respectively; there was no difference within the alcohol groups.

### Primary variables

The statistical significance of the regression coefficients of the mixed models for all four primary variables is presented in Table 1.

**Table 1:** *p* values of the coefficients of the primary variables in the mixed model

Parameter	Treatment	Baseline value	Treatment day	Time of measurement	Interaction treatment/time of measurement
Headache	0.32295	<0.0001^a^	0.99001	0.11912	0.70058
Weakness	0.00038^a^	0.00109^a^	0.49429	0.05087	0.05634
Salivation	0.07444	<0.0001^a^	0.20777	0.00012^a^	0.54592
Motor coordination	<0.0001^a^	0.03532	0.14744	0.00002^a^	0.38169

^a^significant at the significance-level 0.0125

**Fig. 1:**
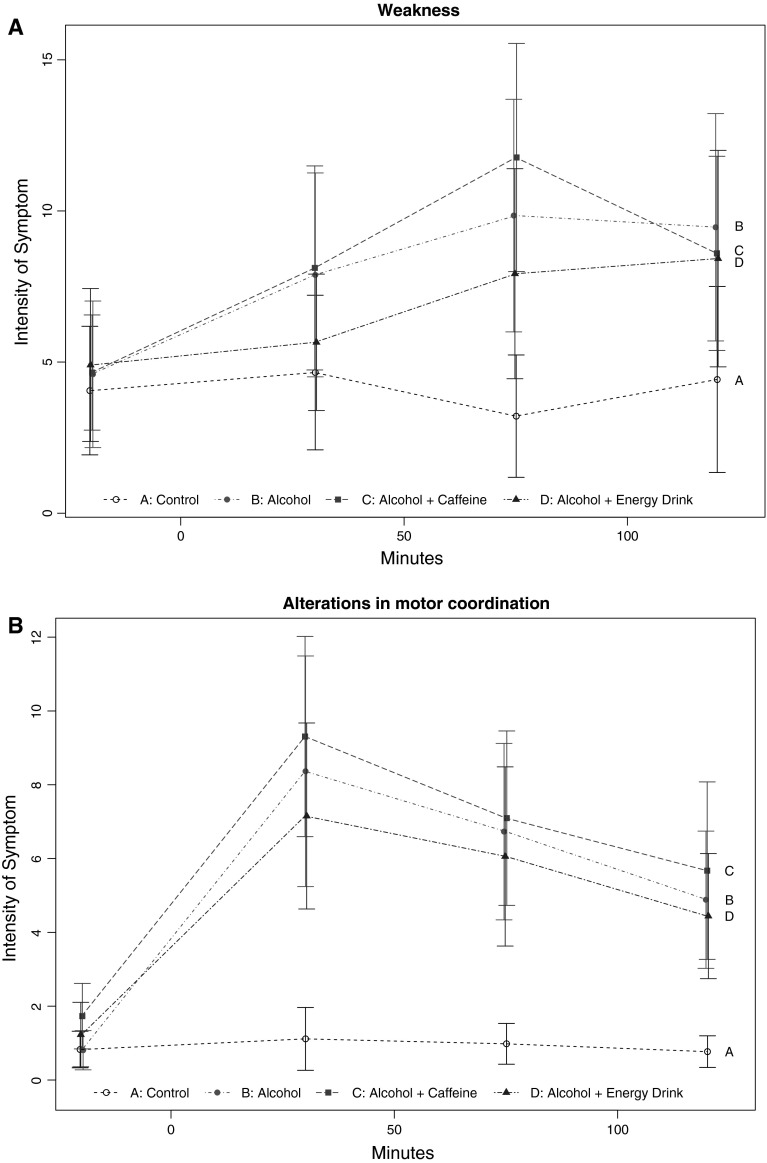
Primary parameter. **a** Weakness shows the intensity of weakness at four different time points (20 min pre-treatment, 30, 90 and 120 min post-treatment). The *black A line* shows the control group. The *red B line* shows the alcohol-only group. The *green C line* shows the alcohol and caffeine group. The *blue D line* shows the alcohol and energy drink group. The *y*-axis only shows the relevant parts oft the total 0–100 scales. There was no difference between the alcohol groups, but a statistically significant difference was observed between the alcohol groups and the non-alcohol group when corrected for multiple testing. **b** Alterations in motor coordination shows the intensity of alterations in motor coordination at four different time points (20 min pre-treatment, 30, 90 and 120 min post-treatment). The *black A line* shows the control group. The *red B line* shows the alcohol-only group. The *green C line* shows the alcohol and caffeine group. The *blue D line* shows the alcohol and energy drink group. The *y*-axis only shows the relevant parts oft the total 0–100 scales. There was no difference between the alcohol groups, but a statistically significant difference was observed between the alcohol groups and the non-alcohol group when corrected for multiple testing (color figure online)

For the variables, weakness and motor coordination, a statistically significant difference between all four treatment groups was observed; in neither of these variables a statistically significant influence of the treatment could be revealed in the sub-analysis of the three groups with alcohol. This indicates that differences between the four treatment groups in weakness and motor coordination were mainly driven due to the linear values recorded for the non-alcohol group (see Fig. 1a, b).

The time of measurement was significant for salivation, motor coordination and the three-group analysis of weakness, but no treatment–time interaction could be shown for any of the four primary variables. The number of the visit reflecting treatment order had no significant influence on any of the models.

### Secondary variables

Eleven of the 14 secondary variables showed an uncorrected significance level of less than 0.05 %. Out of these, five revealed significant differences between the four treatment groups when corrected for multiple testing (see Table 2), i.e. dizziness, alterations in sight, alterations in walking, agitation and alterations in speech.

**Table 2:** *p* values of the coefficients of the secondary variables in the mixed model

Symptom	Treatment	Baseline value	Treatment day	Time of measurement	Interaction treatment/time of measurement
Tiredness	0.01911	<0.0001	0.22595	<0.0001	0.06376
Dizziness	**<0.0001**	**0.53792**	**0.41346**	**<0.0001**	**0.00034**
Tremor	0.01814	<0.0001	0.76371	0.01015	0.51661
Tension	0.50898	<0.0001	0.17191	0.39731	0.56081
Nausea	0.09716	0.00002	0.00566	0.68368	0.35981
Perspiration	0.00523	<0.0001	0.30909	<0.0001	0.54631
Alterations in sight	**<0.0001**	**0.00030**	**0.00701**	**<0.0001**	**0.00312**
Tachycardia	0.04811	0.00002	0.08747	0.30345	0.05890
Breathing difficulty	0.01281	<0.0001	0.45282	0.19071	0.77901
Alterations in walking	**<0.0001**	**0.00222**	**0.82269**	**<0.0001**	**0.00706**
Agitation	**0.00069**	**<0.0001**	**0.12238**	**0.00012**	**0.27364**
Alterations in hearing	0.00614	0.00138	0.17064	0.26217	0.09431
Alterations in speech	**<0.0001**	**0.00986**	**0.09885**	**<0.0001**	**0.22069**
Well-being	0.09532	<0.0001	0.91220	0.04818	0.72580

Bold remain significant when corrected for multiple testing

**Fig. 2:**
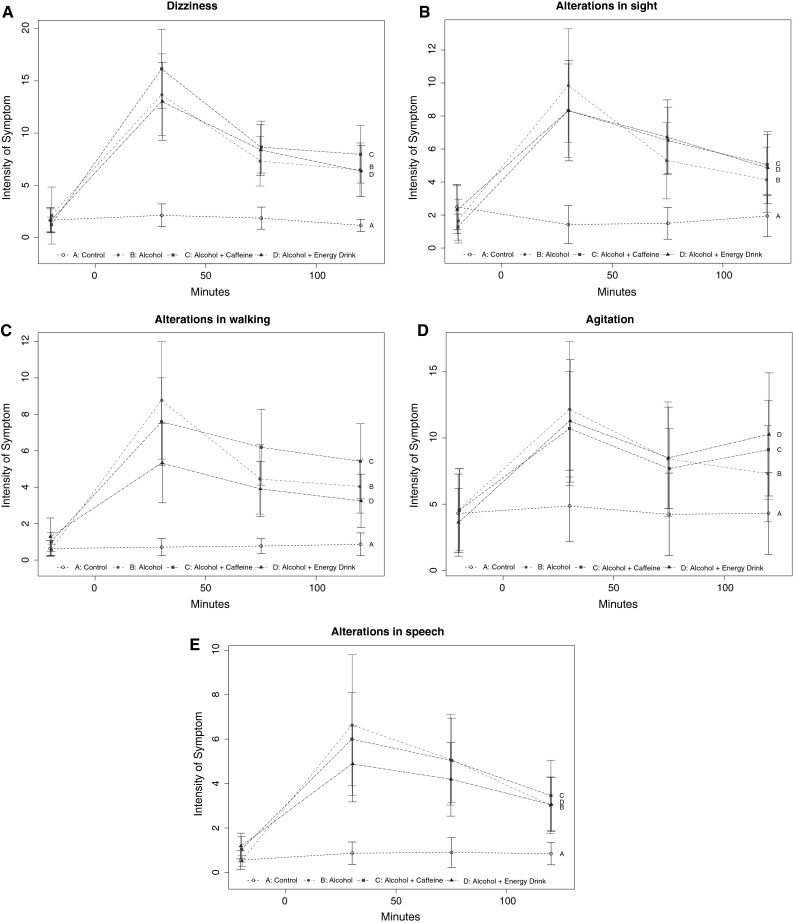
Secondary parameter. **a** Dizziness shows the intensity of dizziness at four different time points (20 min pre-treatment, 30, 90 and 120 min post-treatment). The *black A line* shows the control group. The *red B line* shows the alcohol-only group. The *green C line* shows the alcohol and caffeine group. The *blue D line* shows the alcohol and energy drink group. The *y*-axis only shows the relevant parts oft the total 0–100 scales. There was a significant difference between the four treatment groups when corrected for multiple testing, but no difference was observed in the sub-analysis of the three alcohol groups. **b** Alterations in sight shows the intensity of alterations in sight at four different time points (20 min pre-treatment, 30, 90 and 120 min post-treatment). The *black A line* shows the control group. The *red B line* shows the alcohol-only group. The *green C line* shows the alcohol and caffeine group. The *blue D line* shows the alcohol and energy drink group. The *y*-axis only shows the relevant parts of the total 0–100 scales. There was a significant difference between the four treatment groups when corrected for multiple testing, but no difference was observed in the sub-analysis of the three alcohol groups. **c** Alterations in walking shows the intensity of alterations in walking at four different time points (20 min pre-treatment, 30, 90 and 120 min post-treatment). The *black A line* shows the control group. The *red B line* shows the alcohol-only group. The *green C line* shows the alcohol and caffeine group. The *blue D line* shows the alcohol and energy drink group. The *y*-axis only shows the relevant parts of the total 0–100 scales. There was a significant difference between the four treatment groups when corrected for multiple testing, but no difference was observed in the sub-analysis of the three alcohol groups. **d** Agitation shows the intensity of agitation at four different time points (20 min pre-treatment, 30, 90 and 120 min post-treatment). The *black A line* shows the control group. The *red B line* shows the alcohol-only group. The *green C line* shows the alcohol and caffeine group. The *blue D line* shows the alcohol and energy drink group. The *y*-axis only shows the relevant parts of the total 0–100 scales. There was a significant difference between the four treatment groups when corrected for multiple testing, but no difference was observed in the sub-analysis of the three alcohol groups. **e** Alteration in speech shows the intensity of alteration in speech at four different time points (20 min pre-treatment, 30, 90 and 120 min post-treatment). The *black A line* shows the control group. The *red B line* shows the alcohol-only group. The *green C line* shows the alcohol and caffeine group. The *blue D line* shows the alcohol and energy drink group. The *y*-axis only shows the relevant parts oft the total 0–100 scales. There was a significant difference between the four treatment groups when corrected for multiple testing, but no difference was observed in the sub-analysis of the three alcohol groups (color figure online)

In the sub-analysis of the three groups with alcohol none of these variables revealed a significant difference between treatments. This indicates that differences between the four treatment groups were mainly driven due to differences with the non-alcohol control group (see Fig. 2a–e).

## Discussion

In our study, findings of a so-called “masking effect” could not be reproduced. First, the parameters Salivation and Headache showed no differences between the alcohol-containing treatment conditions and the placebo condition despite our larger population sample. With regards to the two other primary parameters perception of Weakness and Impairment of Motor Coordination, our data confirmed a significant effect of alcohol—that also remained robust following correction for multiple comparisons. However, when the subset of alcohol-containing treatment conditions were analysed in a three-group model—that excluded the overriding effects of alcohol—we were not able to detect a masking effect of the combination of energy drink or caffeine and alcohol compared to the alcohol-only treatment condition, even without correcting for multiple comparisons.

The use of VAS as a method to evaluate subjective feelings has a long tradition as “comparisons can be achieved with greater sensitivity than with semantic phrases or numeric rating scales” (Aitken 1969). Starting with the measurement of feelings and moods (Bond and Lader 1972; Aitken 1969; Folstein and Luria 1973), VAS were also used for rating physiological signs of emotions and adapted increasingly for clinical use (Greenwood et al. 1975). For their study, Ferreira had added five scales to the original 13 items of the Bond and Lader VAS without prior validation as a measure of intoxication (Ferreira et al. 2006; Bond and Lader 1972). This somatic symptom scale, which was also applied in the current study, may therefore not adequately assess the ‘mental’ experience of intoxication—but was chosen to partially replicate the Ferreira study (Ferreira et al. 2006). In a recent review of the literature (Hjermstad et al. 2011), VAS scales were compared to other methods of rating and confirmed as valid and the most frequently used option. The conditions of its use rather than the type of scale determined the quality of VAS-based methods (Hjermstad et al. 2011). Accordingly, the current study used well-established anchor descriptors (analogous to Ferreira et al. 2006) and a written instruction that was orally checked for comprehension.

When the statistical analysis of the data obtained by the VAS did not include corrections for multiple comparisons, 13 out of the investigated 18 parameters in the present study differed significantly between alcohol and non-alcohol containing drinks. Ferreira had detected effects of alcohol in fewer parameters, failing to detect differences in parameters such as Tremor, Perspiration, Tachycardia, Breathing difficulty, Agitation and Alteration in hearing. In contrast, Ferreira found significant differences in Headache and Salivation, whereas our data did not demonstrate differences between the alcohol and the non-alcohol group in these parameters. When corrected for multiple comparisons—as is imperative in this methodology—five of our parameters still remained significant with regards to alcohol effects. Unfortunately, this comparison cannot be performed with Ferreira’s paper, as this information is not given in his data. However, the overall broad overlap of results in response to alcohol between our study and that of Ferreira again supports the validity of the VAS as an assessment tool, and confirms the sensitivity of this tool in our population sample.

In the Ferreira analysis, the differences in perception of salivation, headache, weakness and impairment of motor coordination were statistically significant between the non-alcohol and the alcohol-only group, but were not significantly different between the non-alcohol and the alcohol combined with energy drink group. As there were no significant effects detected in objective measures of visual reaction time or motor coordination, the authors interpreted these findings as a “masking effect”.

The differentiation between primary and secondary outcome parameters in our study was based on the assumption that the four parameters that described so-called “masking effects” in the Ferreira paper were a suitable subset for primary outcome parameters. As these primary outcome parameters failed to confirm a “masking effect” of combined ingestion of energy drink or caffeine with alcohol, and as this differentiation in primary and secondary outcome parameters was somewhat arbitrary, we also performed a statistical analysis of the other 14 symptoms measured by the VAS.

For half of these parameters, the two studies were in agreement: regarding tension and nausea neither Ferreira et al. nor we could find any significant differences (even without correction for multiple testing). Ferreira et al. showed a significant difference in the parameters Tiredness, Dizziness*, Alterations in Sight*, Alterations in Walking*, Alterations in Speech* between alcoholic and non-alcoholic drinks. We also found a similar difference between alcoholic and non-alcoholic drinks for these parameters, in four out of the five, even after correction for multiple testing (only Tiredness lost significance after correction for multiple testing).

For the other seven parameters, the two studies were not in agreement: regarding the parameters Tremor, Perspiration, Tachycardia, Breathing Difficulty, Agitation*, and Alterations in Hearing Ferreira et al. could not find a significant difference between all groups. In contrast, in our study we found a significant difference between the alcoholic and non-alcoholic drinks, which, however, was lost after correction for multiple testing for all parameters except Agitation.

As also demonstrated in Table 3, the agreement of any given parameter between the two studies strongly depended on the results of the correction for multiple testing. These data underline the importance of this statistical correction to reduce the risk of false positive findings. Out of the seven parameters (two primary and five secondary) that remained statistically significant after correction for multiplicity, six had also been described by Ferreira, resulting in an agreement of 86 %! In contrast, out of the six parameters that lost their statistical significance by correction for multiple testing, only one had also been described by Ferreira, resulting in an agreement of only 17 %! In other words, correction of multiplicity increased the likelihood for agreement between the two studies fivefold. None of these parameters demonstrated a “masking effect” of combined ingestion of energy drink or caffeine with alcohol. This lack of a “masking effect” was particularly well presented in the graphical display of the original data (Figs. 1, 2) that showed a clear separation between the alcohol and the non-alcohol containing drinks with no discernible effect of the addition of energy drink or caffeine across a range of subjective parameters.

**Table 3:** Agreement between the Ulbrich and the Ferreira Studies´ variable parameters

	Ulbrich YES	Ulbrich NO
4 Primary parameters		
Ferreira YES	**Weakness**, **impairment of motor coordination**	Dry mouth headache
14 Secondary parameters		
Ferreira YES	Tiredness,** Dizziness**,** Alterations in sight**,** Alterations in walking**,** Alterations in speech**	Well-being
Ferreira NO	Tremor, Perspiration, Tachycardia, Breathing difficulty, **Agitation**, Alterations in hearing	Tension, nausea

Bold parameters remained statistically significant after correction for multiplicity

In conclusion, although testing twice the number of participants at the lower dose of alcohol, rendering our design more sensitive to the detection of such effects, this within-subjects study failed to reproduce results from Ferreira’s publication in 2006 with regards to a so-called “masking effect” when combining caffeine or energy drink with alcohol compared to alcohol-only consumption. As we did not perform objective measures in this study, our results do not allow conclusions regarding other parameters such as motor coordination and visual reaction time. These results thus add to other evidence reviewed by the UK Committee on Toxicity (2012) (http://cot.food.gov.uk/pdfs/tox201210.pdf) that the masking effect of energy drinks cannot be confirmed by currently available data.
